# HSPG-binding peptide Pep19-2.5 is a potent inhibitor of HPV16 infection

**DOI:** 10.1128/aac.01575-24

**Published:** 2025-01-14

**Authors:** Snježana Mikuličić, Annika Massenberg, Tatjana Döring, Klaus Brandenburg, Thorsten Lang, Luise Florin

**Affiliations:** 1 Institute for Virology, University Medical Center of the Johannes Gutenberg-University Mainz 39068 , Mainz, Germany; 2 University of Bonn, Faculty of Mathematics and Natural Sciences, Life & Medical Sciences (LIMES) Institute 88772 , Bonn, Germany; 3 Brandenburg Antiinfektiva GmbH, Research Center Borstel-Leibniz-Lung Center 28413 , Borstel, Germany; 4 Research Center for Immunotherapy (FZI), University Medical Center of the Johannes Gutenberg-University Mainz 39068 , Mainz, Germany; IrsiCaixa Institut de Recerca de la Sida, Barcelona, Spain

**Keywords:** HPV16, human papillomavirus, synthetic anti-lipopolysaccharide peptide, SALP, Peptide 19-2.5, Pep19-2.5, Aspidasept, heparan sulfate proteoglycans (HSPGs), virus entry

## Abstract

Peptide-based therapeutics are gaining attention for their potential to target various viral and host cell factors. One notable example is Pep19-2.5 (Aspidasept), a synthetic anti-lipopolysaccharide peptide that binds to heparan sulfate proteoglycans (HSPGs) and has demonstrated inhibitory effects against certain bacteria and enveloped viruses. This study explores, for the first time, the effectiveness of Pep19-2.5 against a non-enveloped virus, using pseudoviruses of the oncogenic human papillomavirus type 16 (HPV16) as a model. HPV16 infects epithelial cells of the skin and mucosa by using multiple cell surface receptors with initial attachment to HSPGs. Pharmacological inhibition with Pep19-2.5 in HeLa and HaCaT cells resulted in a concentration-dependent reduction of HPV16 PsV infection, with near-complete blockade observed at higher concentrations. The half-maximal inhibitory concentration (IC_50_) was determined to be 116 nM in HeLa cells and 183 nM in HaCaT cells, highlighting its potent antiviral activity. Our results demonstrate that Pep19-2.5 not only inhibits HPV16 PsV binding to the cell surface but also significantly reduces infection when administered post-binding. Imaging analyses revealed Pep19-2.5-dependent release of large cell-associated crowds of viral particles, suggesting interference with the transfer to secondary receptor molecules. This was corroborated by the effectiveness of Pep19-2.5 in an HSPG-negative cell line, indicating that the peptide disrupts virus binding to both primary and secondary interaction partners. Based on these findings, we propose that the antimicrobial effect of Pep19-2.5 is not limited to HSPG-dependent infections. Additionally, Pep19-2.5 may be a valuable tool for dissecting specific steps in the viral entry process.

## INTRODUCTION

Antiviral peptides have emerged as promising therapeutic targets against a wide range of DNA and RNA viruses, including cytomegalovirus (CMV), herpes simplex virus type 1 (HSV-1) and type 2 (HSV-2), adenovirus, rotavirus, poliovirus, respiratory syncytial virus (RSV), human immunodeficiency virus (HIV), influenza virus, hepatitis B virus (HBV), hepatitis C virus (HCV), and the dengue, chikungunya, and Zika viruses, targeting various stages of viral infection (1). A novel class of inhibitors known as synthetic anti-lipopolysaccharide peptides (SALPs) has been suggested as suitable broad-spectrum antiviral and anti-inflammatory agents as they exhibit low cytotoxicity and effectively block the attachment, and as a result, the infection of human pathogenic viruses (2, 3). Among these inhibitors, Peptide 19-2.5 (also known as Aspidasept or Pep19-2.5) has attracted attention for its ability to inhibit the entry of various enveloped viruses, including hepatitis B, hepatitis C, and human immunodeficiency virus (3). This is achieved through its binding to heparan sulfate proteoglycans (HSPGs) and sialic acids on the plasma membrane. Studies suggest that the positively charged amino acids within Pep19-2.5 compete with the virus for the binding to the negatively charged plasma membrane components (2, 3). In addition, it has been demonstrated that Pep19-2.5 possesses anti-septic properties *in vitro* and *in vivo* by reducing the production of pro-inflammatory cytokines (4, 5). The influence of Pep19-2.5 on non-enveloped viruses has not been investigated so far.

Human papillomaviruses (HPVs) are small, non-enveloped DNA viruses that cause various malignant tumors, like vaginal, vulval, penal, and head and neck tumors. HPVs are classified into low-risk types, which cause benign lesions, and high-risk types such as HPV16, which can immortalize cells and lead to cancer (6, 7). HPVs structurally consist of the major capsid protein L1, the minor capsid protein L2, and the viral genome (8, 9). Due to the challenges in propagating HPV in cell culture, pseudoviruses (PsVs) are a widely used tool to investigate virus entry. These PsVs contain capsid proteins and a pseudogenome encoding a reporter gene. In this study, we use HPV16 PsVs containing a luciferase reporter gene that is controlled by HPV’s long control region (LCR), as described previously (10, 11).

HPV infects mitotically active epithelial cells in the basal layer of epithelium (12). The primary attachment sites on the cell surface are negatively charged HSPGs which interact with positively charged lysine residues of the L1 protein (13–19). Other glycosaminoglycans (GAGs), such as chondroitin sulfates, can also serve as primary cell surface receptors for HPV, although they play a less important role in infection (20, 21). The interaction between HPV and heparan sulfate is crucial for virus entry into the infectious pathway as it induces conformational changes (structural activation) of the capsid, lowers affinity to HSPGs, and enables both enzymatic processing of the capsid proteins and transfer to second receptor complex (22–26). In an alternative model, the transfer of the viral particles from primary attachment sites to the second receptor is enabled by the proteolytic cleavage of HSPGs which results in a transient release of soluble HSPG-decorated virions before binding to secondary receptor molecules (16).

Subsequently, a virus-bound second receptor complex is formed, which includes components such as integrins, growth factor receptors, and the tetraspanin CD151 (27, 28). After internalization, HPV16 PsVs are transported in a transport vesicle toward the nucleus. Nuclear entry requires a mitotic cell cycle, which allows the attachment of the viral DNA to the mitotic chromatin and the incorporation into newly formed PML nuclear bodies, where viral gene transcription occurs (12, 29–31).

In this study, we examine the impact of Pep19-2.5 on the non-enveloped virus HPV16 and its infection of epithelial cells. We determine the inhibitory potential of the heparan sulfate-binding Pep19-2.5 on infection and delineate its mechanism of action during the initial steps of HPV entry.

## RESULTS

### Pep19-2.5 effectively inhibits HPV16 PsV infection in HeLa and HaCaT cells

**Fig 1:**
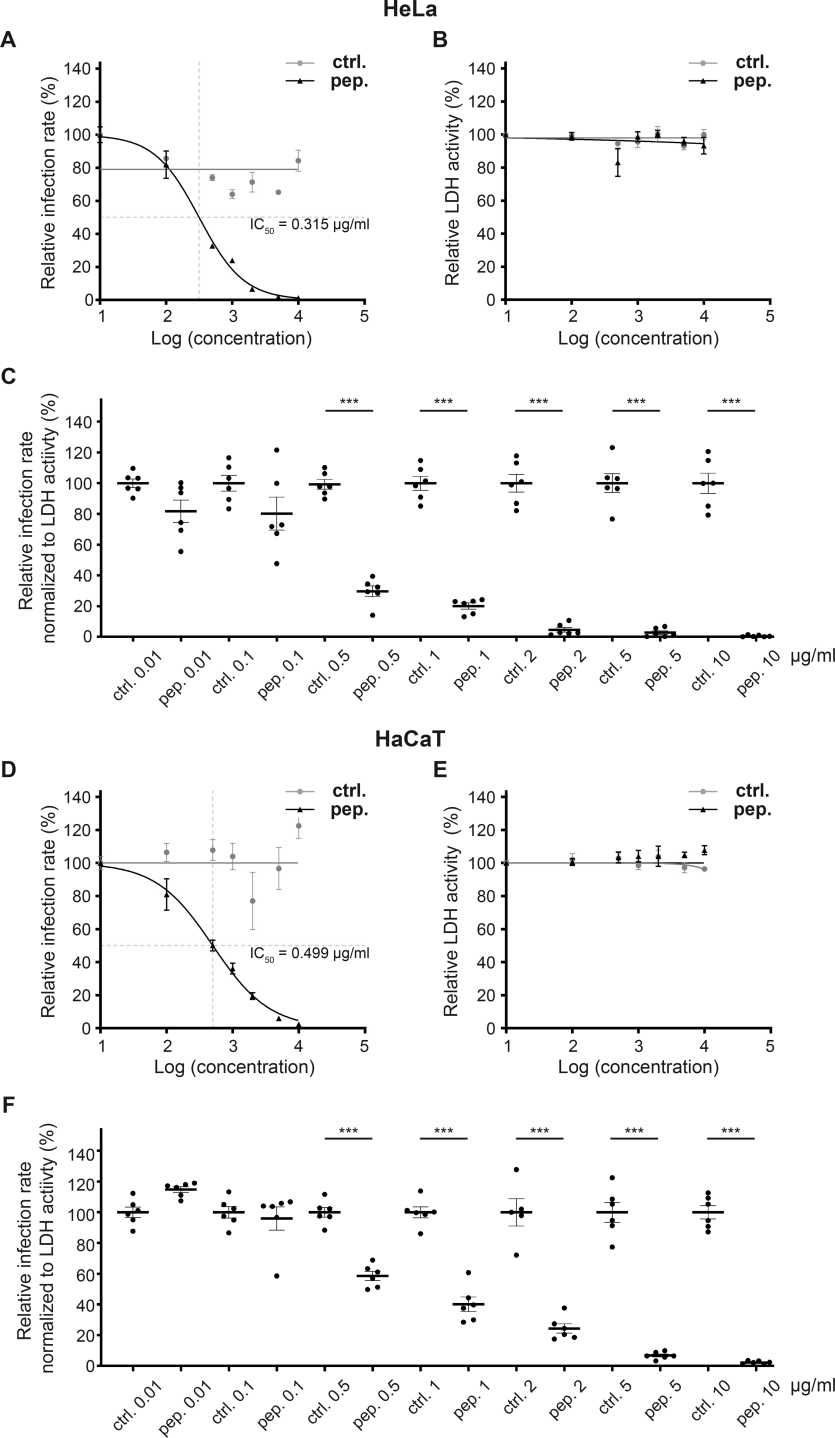
Pep19-2.5 is a potent inhibitor of HPV16 PsVs infection in HeLa and HaCaT cells. HeLa cells (**A–C**) or HaCaT cells (**C–F**) were pre-treated with either control peptide (ctrl.) or Pep19-2.5 (pep.) at concentrations ranging from 0.01 to 10 µg/mL for 1 hour. Subsequently, the cells were infected with HPV16 PsVs encoding for a luciferase for quantification of infection rate. Pseudovirus infection assays (**A, D**) and LDH measurements (**B, E**) were conducted 24 hours later. (**A**) Dose-response curve of control peptide (in gray) and Pep19-2.5 (in black) on infection in HeLa cells. The half-maximal inhibitory concentration (IC_50_) was calculated to be 0.315 µg/mL with a 95% confidence interval (CI) of 0.258–0.380 µg/mL. (**B**) Dose-response curve of control peptide (in gray) and Pep19-2.5 (in black) on cellular LDH measurements in HeLa cells. (**A, B**) The infection rate or LDH measurements are presented relative to the lowest peptide concentration (0.01 µg/mL), with the mean set at 100%. (**C**) Infection rate in HeLa cells normalized to the LDH for different peptide concentrations. For each concentration, the mean of the normalized infection rate for the control peptide was set at 100%. Statistical differences between the groups (*n* = 6) were analyzed using Welch’s *t* test (*P* = 0.0569 for ctrl. 0.01 vs pep. 0.01, *P* = 0.1395 for ctrl. 0.1 vs pep. 0.1, and *P* < 0.0001 for all other comparisons). (**D**) Dose-response curve of control (in gray) or Pep19-2.5 (in black) on infection in HaCaT cells. The IC_50_ was calculated as 0.499 µg/mL (95% CI = 0.399–0.613 µg/mL). (**E**) Dose-response curve of control (in gray) or Pep19-2.5 (in black) on cellular LDH measurements. (**D, E**) The infection rate or LDH measurements are presented relative to the lowest peptide concentration (0.01 µg/mL) with the mean set at 100%. (**F**) Infection rate in HaCaTs normalized to the LDH for different peptide concentrations. For each concentration, the mean of the normalized infection rate for the control peptide is set at 100%. Statistical differences between the groups (*n* = 6) were analyzed using Welch’s *t* test (*P* = 0.0056 for ctrl. 0.01 vs pep. 0.01, *P* = 0.6503 for ctrl. 0.1 vs pep. 0.1, *P* = 0.0005 for ctrl. 2 vs pep. 2, and *P* < 0.0001 for all other comparisons). (**A–F**) Significance stars are displayed to indicate significant infection inhibition. The data are presented as mean ± SEM.

To evaluate the impact of Pep19-2.5 on infections by the non-enveloped papillomavirus type 16, we initially performed infection assays with increasing concentrations of both the control peptide and Pep19-2.5 to investigate the effects on infection rate and cell viability (lactate dehydrogenase [LDH] activity) in HeLa (Fig. 1A through C) and HaCaT (Fig. 1D through F) cells. Examination of the HPV16 PsV infection rate in cells pre-treated with the peptide and subsequently infected revealed comparable results for both cell lines with an indeterminable IC_50_ for the control peptide, while for Pep19-2.5, the IC_50_ was determined to be 0.315 µg/mL (116 nM) for HeLa and 0.488 µg/mL (183 nM) for HaCaT cells (Fig. 1A and D). Additionally, we assessed the cellular LDH activity to test for putative cytotoxic effects of the peptides. Cytotoxic effects are accompanied by a loss of membrane integrity and LDH. No significant reduction in LDH activity of cell lysates was detectable for either peptide at any concentration in both cell lines (Fig. 1B and E) suggesting that no cytotoxic effect was induced. Infection rates normalized to the LDH measurements and presented relative to the respective controls are shown in Fig. 1C and F, to enable comparability to subsequent experiments and statistics of the dose response. The calculations indicate that higher concentrations of Pep19-2.5 progressively reduce the infection rate, with a significant reduction observed at concentrations as low as 0.5 µg/mL in both cell lines. Furthermore, a Pep19-2.5 concentration of 2 µg/mL and 5 µg/mL almost completely inhibited infection in HeLa and HaCaT cells, respectively.

### Pep19-2.5 hinders PsV-cell surface binding

**Fig 2:**
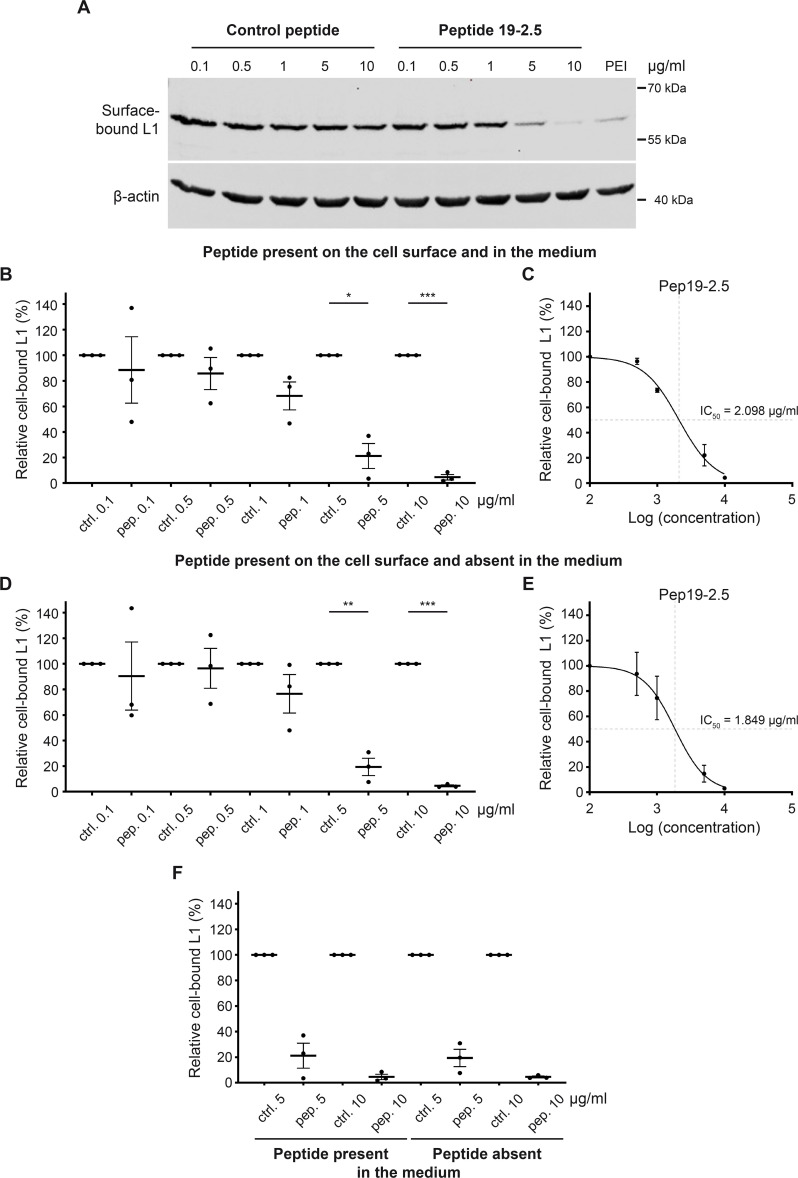
Pep19-2.5 hinders PsV-cell surface binding. (A–C) A confluent layer of HeLa cells was pre-treated with either control (ctrl.) or Pep19-2.5 (pep.) at concentrations ranging from 0.1 to 10 µg/mL for 1 hour. Subsequently, the cells were infected with HPV16 PsVs for 2 hours at 4°C to minimize virus internalization. The cells were then washed three times with phosphate-buffered saline (PBS) and harvested for Western blot (WB). (**A**) WB showing surface-bound L1 detected with L1-specific antibody (312-F). β-Actin was used as a loading control. (**B**) Quantification of the surface-bound L1 normalized to the loading control from WB shown in (**A**). For each concentration, the mean of the assessed L1 for the control peptide was set at 100%. Statistical differences between the groups (*n* = 3) were analyzed using Welch’s *t* test (*P* = 0.7035 for ctrl. 0.1 vs pep. 0.1, *P* = 0.3741 for ctrl. 0.5 vs pep. 0.5, *P* = 0.1015 for ctrl. 1 vs pep. 1, *P* = 0.0149 for ctrl. 5 vs pep. 5, and *P* = 0.0005 for ctrl. 10 vs pep. 10). (**C**) Dose-response curve of Pep19-2.5 on PsV-cell surface binding with peptide present on the cell surface and in the medium (without medium change) before PsVs addition. Surface-bound PsVs are presented relative to the lowest Pep19-2.5 concentration (0.1 µg/mL) with the mean set at 100%. (D, E) A confluent layer of HeLa cells was treated with the peptides as in (A–C), but with peptide present on the cell surface and absent in the medium (with medium change) before PsVs addition. (**D**) Quantification of the surface-bound L1 normalized to the loading control (WB not shown). For each concentration, the mean of the assessed L1 for the control peptide was set at 100%. Statistical differences between the groups (*n* = 3) were analyzed using Welch’s *t* test (*P* = 0.7550 for ctrl. 0.1 vs pep. 0.1, *P* = 0.8443 for ctrl. 0.5 vs pep. 0.5, *P* = 0.2601 for ctrl. 1 vs pep. 1, *P* = 0.0069 for ctrl. 5 vs pep. 5, and *P* < 0.0001 for ctrl. 10 vs pep. 10). (**E**) Dose-response curve of Pep19-2.5 on PsV-cell surface binding with medium change before PsVs addition. Surface-bound PsVs are presented relative to the lowest Pep19-2.5 concentration (0.1 µg/mL) with the mean set at 100%. (**F**) Comparison of experimental conditions: without medium change before PsVs addition (peptide present on the cell surface and in the medium) and with medium change (peptide is present on the cell surface and absent in the medium). Statistical differences between the groups (*n* = 3) were analyzed using Welch’s *t* test (*P* = 0.8884 for pep. 5 present vs pep. 5 absent and *P* = 0.9928 for pep. 10 present vs pep. 10 absent). (A–F) The data are presented as mean ± SEM.

Following the observation of a significant decrease in the infection rate by Pep19-2.5, we investigated the underlying antiviral mechanism. First, we investigated how Pep19-2.5 affects virus binding to the cell surface (Fig. 2). Here, non-polar HeLa cells were used to enable PsV-cell binding to the apical membrane of a confluent cell layer to avoid PsV binding to the culture dish. Polyethyleneimine (PEI), a previously documented polycationic agent recognized for its inhibition of HPV-cell binding, was used as a control (32). As expected, PEI almost blocked PsV-cell binding (see remaining weak L1 band in Fig. 2A). The levels of L1 in the cell lysates treated with Pep19-2.5 decreased proportionally to the peptide concentration, reaching at the largest concentration levels even below that observed in PEI-treated cells (Fig. 2A). In conclusion, a concentration of 10 µg/mL of Pep19-2.5 caused almost a complete block of PsV-cell binding, with the half-maximal inhibitory concentration (IC_50_) of 2.098 µg/mL (773 nM) (Fig. 2B and C).

To ensure that potential excess peptides in the medium did not exert additional inhibitory effects by directly binding to PsVs, we changed the cell culture medium before adding the PsVs. The results showed comparable inhibition curves for both treatments (Fig. 2B through E), with an IC_50_ value of 1.849 µg/mL (681 nM) with Pep19-2.5 present on the cell surface and absent in the medium (Fig. 2E). A statistical direct comparison of the two conditions revealed no significant differences with and without the peptide present in the medium (Fig. 2F). Therefore, this experiment confirms that the peptide hinders infection by binding to the cells and not to PsVs.

Comparing the IC_50_ values for infection rate (Fig. 1) and binding (Fig. 2) reveals that approximately 6.5 times higher concentrations of the peptide are necessary to impede HPV binding to the cells. This difference becomes obvious when comparing the effects of a 1 µg/mL of Pep19-2.5 on infection and PsV-cell binding. While at that concentration, infection is strongly inhibited, the effect on PsV-cell association is only moderate. This suggests that Pep19-2.5 has an additional role in hindering PsV infection beyond its impact on PsV-cell binding.

### Pep19-2.5 is still active after HPV16 PsV-cell binding but does not affect PsV-CD151 association

**Fig 3:**
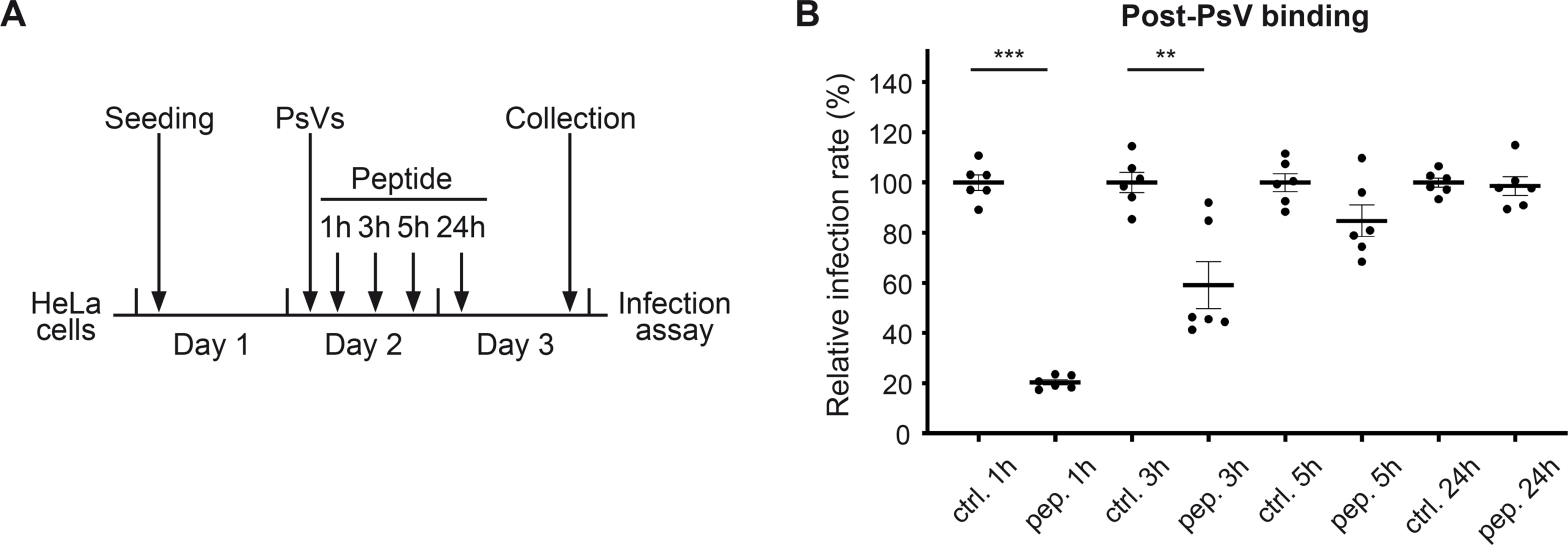
Pep19-2.5 is still active after PsV-cell binding. (**A**) Scheme of the experimental procedure. HeLa cells were exposed to the HPV16 PsVs and subsequently treated with 1 µg/mL of the control (ctrl.) or Pep19-2.5 (pep.).The luciferase assay was performed 24 hours after infection. (**B**) The infection rate normalized to the LDH for different time points. At each time point, the mean of the infection rate for the control peptide is set at 100%. Statistical differences between the groups (*n* = 6) were analyzed using Welch’s *t* test (*P* < 0.0001 for ctrl. 1 hour vs pep. 1 hour, *P* = 0.0054 for ctrl. 3 hours vs pep. 3 hours, *P* = 0.0682 for ctrl. 5 hours vs pep. 5 hours, and *P* = 0.7525 for ctrl. 24 hours vs pep. 24 hours). The data are presented as mean ± SEM.

To evaluate if Pep19-2.5 remains effective after virus exposure, we adjusted our experimental setup (Fig. 3A). After exposing cells to HPV16 PsVs, we treated them with 1 µg/mL peptide, a concentration able to significantly reduce infection rates, for various durations and analyzed 24 hours after PsVs exposure (Fig. 3A). Remarkably, there was an 80% decrease in the infection rates when Pep19-2.5 was administered 1 hour after virus addition (Fig. 3B), mirroring the reduction observed with the same peptide concentration in cells pre-treated with the peptide (as demonstrated in Fig. 1C). Extending the time interval between PsVs exposure and peptide treatment resulted in a decreased suppression of infection rates. Administering the peptide 5 hours after virus exposure had no noticeable impact on infection rates. When administered after PsVs binding, Pep19-2.5 could potentially affect HPV’s interaction with a secondary receptor component or other molecular interactions essential for subsequent steps in infection.

**Fig 4:**
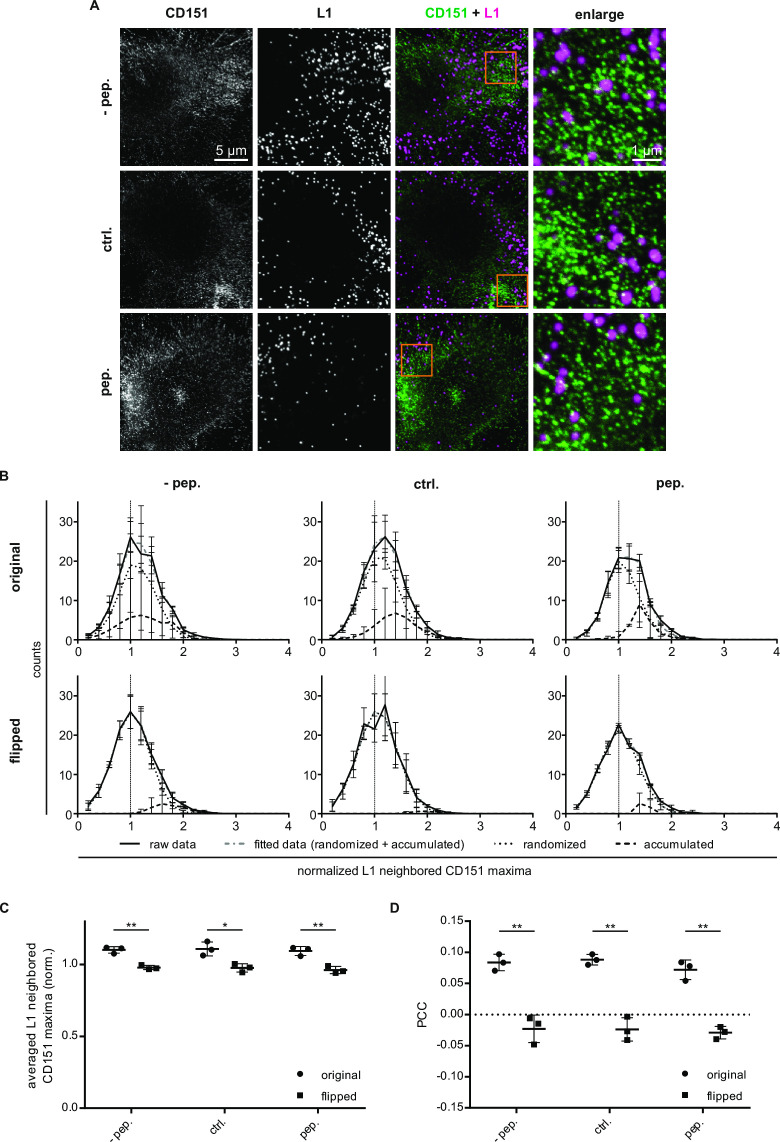
Analysis of CD151 crowding at PsV-binding sites. (**A**) HeLa cells were incubated for a total of 4 hours with PsVs. After 1 hour, they were additionally incubated either without any peptide (−pep.), with 1 µg/mL of the control peptide (ctrl.), or with Pep19-2.5 (pep.). Then, cells were fixed and surface-stained for L1 (magenta) and CD151 (green). *x*,*y*-scans of L1 in the confocal and CD151 in the STED channel were recorded at the basal membrane. Orange boxes mark enlarged views of the overlays shown to the right. (**B**) Analysis of the normalized L1 neighbored CD151 maxima (for details see Materials and Methods). At every L1 position, a circular 925 nm ROI was positioned, the CD151 maxima were counted, and the value was normalized to the CD151 average maxima density. The number of PsVs (defined by the L1 signal) is plotted in absolute counts vs the normalized CD151 maxima counts. At a randomized relationship between L1 and CD151 (horizontally and vertically flipped images), the analysis is supposed to yield a Gaussian-like distribution is supposed to peak at the value of 1. If CD151 maxima at PsV locations are more abundant than random, the distribution becomes right shifted, as it contains two Gaussian distributions. Upper panel: original data showing right-shifted distributions. For determining the fraction of PsVs locating at CD151 crowds, the distribution was decomposed into two Gaussian distributions (dotted lines mark randomized, dashed lines mark the pool of PsVs at accumulated CD151 (crowds), and gray dotted-dashed lines are the sum of both populations). The size of the crowded fractions ranges on average between 21% and 34%. Lower panel: flipped data. The fitted Gaussians peak at 1. The size of the crowded fractions ranges on average between 1% and 7%. (**C**) Same data as in (**B**), showing the average of the normalized L1 neighbored CD151 maxima. Statistical differences between the groups (*n* = 3) were analyzed using Welch’s *t* test (*P* = 0.0027 for −pep. original vs flipped, *P* = 0.0232 for ctrl. original vs flipped, *P* = 0.0051 for pep. original vs flipped, *P* = 0.8453 for ctrl. vs pep. original, *P* = 0.7432 for ctrl. vs pep. original, *P* = 0.9096 for ctrl. vs pep. flipped, *P* = 0.5371 for ctrl. vs pep. flipped). (**D**) Pearson correlation coefficient (PCC) in the ROI between the L1 and CD151 original and flipped (randomized) images. Statistical differences between the groups (*n* = 3) were analyzed using Welch’s *t* test (*P* = 0.0042 for −pep. original vs flipped, *P* = 0.0034 for ctrl. original vs flipped, *P* = 0.0015 for pep. original vs flipped, *P* = 0.6470 for ctrl. vs pep. original, *P* = 0.2136 for ctrl. vs pep. original, *P* = 0.9628 for ctrl. vs pep. flipped, *P* = 0.6924 for ctrl. vs pep. flipped). (B, C) Values are given as mean ± standard deviation (SD) (*n* = 3 from three biological replicates, including 15 images per condition and replicate).

At the cell membrane, the tetraspanin CD151 organizes in clusters that enrich at virus-binding sites and is crucial for virus internalization (33–35). Utilizing super-resolution (STED) microscopy, we studied whether Pep19-2.5 has any effect on the organization of CD151 clusters at PsV-binding sites. Sub-confluent cells were incubated with PsVs for 4 hours, and 1 hour after PsVs addition, either the control peptide or Pep19-2.5 was added at 1 µg/mL. This concentration was particularly interesting because it potently inhibits infection while having only a small effect on PsV-cell binding. After fixation, immunostaining was performed for CD151 and the major capsid protein L1 of the PsVs (Fig. 4A). We studied the basal cell membrane, allowing for recording in one image (in the focal plane) a large area of the cell membrane. We determined the positions of PsVs (defined by L1 immunostaining), counted, at the PsV positions, the number of CD151 maxima in a circular region of interest (ROI) (925 nm diameter), and normalized the counts to the average CD151 maxima density in the image. Hence, if at PsV locations the density of CD151 maxima is equal to the average density, we obtain an average value of 1, and a larger value if CD151 is enriched.

In the following, we refer to the normalized CD151 counts as L1 neighbored CD151 maxima (Fig. 4B). To differentiate between specific association in original images and random background association, we performed the same analysis after flipping the CD151 image horizontally and vertically (in the following referred to as flipped, Fig. 4B, lower panel). In flipped images, the most likely count of neighbored maxima is the average CD151 maxima density, which results in a normal distribution peaking at 1, and the average normalized maxima density is close to 1 (Fig. 4C). In the original images, the distribution is right shifted (Fig. 4B, upper panel), and the average maxima density is elevated (Fig. 4C). Flipping also diminishes the Pearson correlation coefficient (PCC) between the L1 and CD151 channel (Fig. 4D), pointing toward a specific association between PsVs and CD151.

The right-shifted distribution results from two populations of PsVs, one with a random number (as in the flipped images) and one with an elevated number of neighbored maxima. Decomposition of the measured data into two Gaussian distributions yields the fraction of the PsVs with an elevated number of neighbored maxima that ranges between 21% and 34% (in flipped images 1–7%). The data show no indication of a Pep19-2.5 effect on the number of neighbored maxima. Hence, Pep19-2.5 has no effect on the availability of CD151 microdomains.

**Fig 5:**
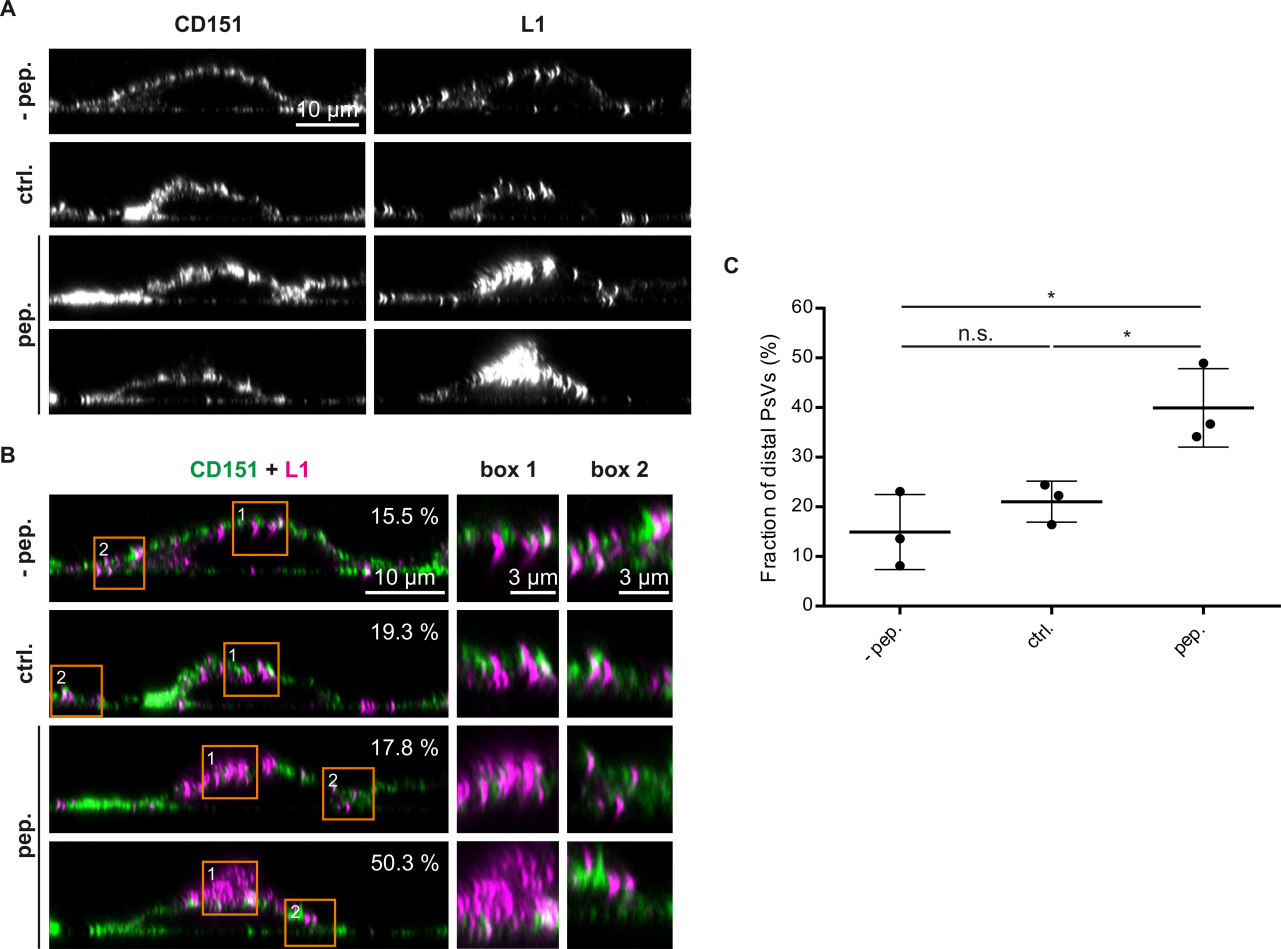
The Pep19-2.5 induces large extracellular PsV accumulations. (**A**) Same preparation as in Fig. 4. In each condition, we imaged by confocal microscopy employing *x*,*z*-scans*/y*,*z*-scans those cells exhibiting the most prominent PsV accumulations. Shown are images of the CD151 and L1 channels of the different conditions (same scaling of the respective channels). We show two examples of the Pep19-2.5 condition to point out that, in the presence of Pep19-2.5, we observe cells resembeling those in the control condition, as well as cells with very large PsV accumulations, which are typically localized at the top of the cell (see also Fig. S1 and S2). (**B**) Same images as in (**A**), auto-scaled, and shown as overlays (L1 in magenta and CD151 in green) with magnified views of the orange boxes. Values in the upper right indicate the fraction of distal PsVs from which in (**C**) we show the average values. (**C**) Cells were outlined (for example, see Fig. S2A) and the background-corrected L1 signal within the outline was subtracted from the total background-corrected L1 signal, yielding the signal of distal PsVs. For details regarding the calculation of the fraction of distal PsVs, see Materials and Methods. Values are given as means ± SD (*n* = 3 from three biological replicates, including 20 images per condition and replicate; for one replicate, we show almost the complete data set in Fig. S1). Statistical differences between the groups (*n* = 3) were analyzed using Welch’s *t* test (*P* = 0.3033 for ctrl. vs pep., *P* = 0.0348 for ctrl. vs pep., *P* = 0.0167 for −pep. vs pep.).

**Fig 6:**
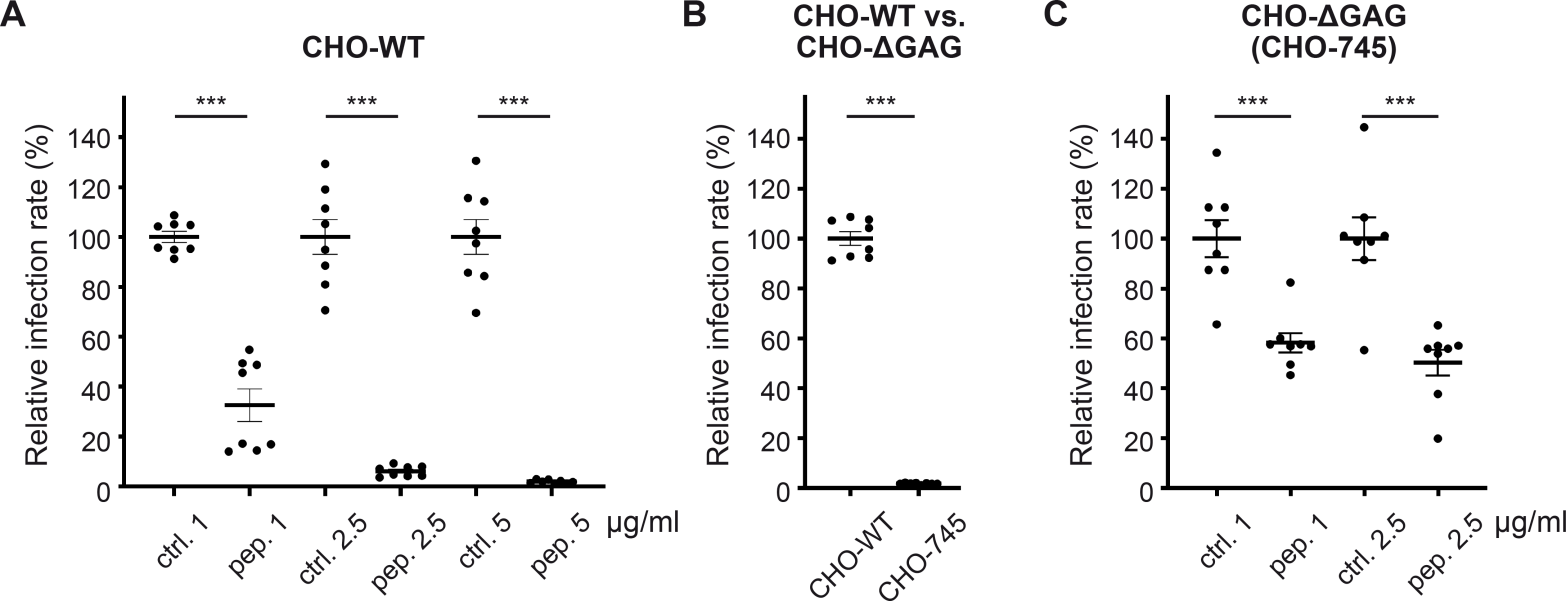
Pep19-2.5 reduces infection rate in CHO cells irrespective of the presence of GAGs. (**A**) CHO-WT cells were pre-treated with either control (ctrl.) or Pep19-2.5 (pep.) at concentrations ranging from 1 to 5 µg/mL for 1 hour. Subsequently, the cells were infected with HPV16 PsVs. The infection rate was normalized to the LDH for different peptide concentrations. For each concentration, the mean of the infection rate for the control peptide is set to 100%. Statistical differences between the groups (*n* = 8) were analyzed using Welch’s *t* test (*P* < 0.0001 for all comparisons). (**B**) CHO-WT and CHO-ΔGAG cells were infected with HPV16 PsVs, and the luciferase assay was performed 24 hours later. The mean of the infection rate normalized to the LDH for the CHO-WT is set at 100%. Statistical differences between the groups (*n* = 8) were analyzed using Welch’s *t* test (*P* < 0.0001). (**C**) CHO-ΔGAG cells were pre-treated with either ctrl. or pep. at 1–2.5 µg/mL for 1 hour. Next, the cells were infected with HPV16 PsVs. Infection rate was normalized to the LDH for different peptide concentrations. For each concentration, the mean of the infection rate for the contr. is set at 100%. Statistical differences between the groups (*n* = 8) were analyzed using Welch’s *t* test (*P* = 0.0005 for ctrl. 1 vs pep. 1, *P* = 0.0004 for ctrl. 2.5 vs pep. 2.5, and *P* = 0.2388 for pep. 1 vs pep. 2.5). (**A–C**) The data are presented as mean ± SEM.

### Pep19-2.5 induces the release of large HPV16 accumulations from the cell surface

Using the same samples, we determine the distribution of PsVs around cells. We observed on a minority of cells large accumulations of viral particles at the apical cellular part, reaching up to several micrometers away from the cell surface (in the following referred to as distal PsVs), present essentially only after treatment with Pep19-2.5 (Fig. 5A and B; Fig. S1). We recorded *x*,*z*/*y*,*z*-confocal micrographs of the cells, specifically searching for cells exhibiting this phenomenon. Quantification of distal PsVs (distally from the CD151 marked plasma membrane, as shown in Fig. S2A) revealed an almost threefold increase when treated with Pep19-2.5 (Fig. 5C). The large distances between PsVs and the cell surface (shown in Fig. S2B) in cells treated with Pep19-2.5 suggest that this peptide induces the release of accumulated viral particles, probably a consequence of disturbed secondary interactions.

### Pep19-2.5 diminishes HPV infection in glycosaminoglycan-deficient cells

Next, we investigated whether additional non-HSPG interactions are affected by Pep19-2.5 and conducted experiments using cells lacking glycosaminoglycans (GAGs), such as heparan sulfate and chondroitin sulfate. Here, Chinese hamster ovary (CHO) cells, encompassing both CHO wild type (CHO-WT) and the GAG-deficient derivatives CHO-745 (referred to as CHO-ΔGAG), were used (36). As expected, CHO-WT cells exhibited a significant concentration-dependent reduction in infection rate (Fig. 6A). Comparing the infection rates between these cells lines, we observed an approximate 98% decrease in infection rate in CHO-ΔGAG compared to CHO-WT (Fig. 6B). This indicates that the lack of HSPGs and CSPGs almost entirely abolishes HPV infection, as previously evidenced (20, 23, 24, 37, 38). However, and in contrast to other HSPG-binding inhibitors such as DSTP (24), even at this minimal infection rate, Pep19-2.5 induces a significant reduction in the absence of HSPGs (Fig. 6C), indicating its ability to hinder HPV binding to other cellular molecules pivotal for HPV infection.

Together, our data suggest that Pep19-2.5 acts as an antiviral peptide by impairing virus binding to HSPGs and additional cell surface receptors.

## DISCUSSION

The study presented here identifies Pep19-2.5 (Aspidasept) as a potent inhibitor of HPV16 PsV infection in epithelial cells, highlighting its potential as an effective antiviral agent against non-enveloped viruses. Using infection assays, we demonstrated that Pep19-2.5 significantly reduced PsV infection in both HeLa and HaCaT cell lines. The inhibition was dose-dependent, with IC_50_ values of 0.315 µg/mL (116 nM) in HeLa cells and 0.499 µg/mL (183 nM) in HaCaT cells. Notably, the control peptide showed no inhibitory effect, confirming the specificity of Pep19-2.5. Additionally, Pep19-2.5 was non-toxic at concentrations up to 10 µg/mL, in line with prior studies confirming its safety (39).

Pep19-2.5 belongs to the class of SALPs that interact with HSPGs and other cell-surface molecules such as N-acetylneuraminic acid, previously shown to inhibit infection by several enveloped viruses, including HIV-1, HSV-1/2, HBV, HCV, and influenza A virus (2, 3). The mechanism of action of Pep19-2.5 varies depending on the virus but can involve both direct binding to viral capsids and interaction with cellular receptors, disrupting key steps in viral entry (2, 3).

Given that HPV is a non-enveloped virus composed of L1 and L2 capsid proteins, which contain stretches of positively charged amino acids (17, 40), it is unlikely that the virus’s capsid surface interacts directly with the similarly charged Pep19-2.5. This is evidenced by experiments showing that HPV-cell binding is comparably inhibited independent of the presence or absence of Pep19-2.5 in the medium. Moreover, previous studies have shown that positively charged molecules can inhibit HPV infection by binding to GAGs (24, 32, 41). Therefore, it is likely that Pep19-2.5 competes with HPV capsid proteins for binding to HSPGs as one of the inhibitory mechanisms. Accordingly, our Western blot experiments, which show a strong reduction in PsVs binding to cells after Pep19-2.5 treatment, demonstrate the role of Pep19-2.5 in blocking the initial binding of HPV16 PsVs to cells. This is in line with a study showing that Pep19-2.5 binds to heparan sulfate moieties thereby inhibiting infection of enveloped viruses that use HSPGs as primary attachment molecules (3).

The significant discrepancy between the IC_50_ values for virus binding and infection inhibition (6.5-fold difference) suggests that Pep19-2.5 may also affect post-binding processes. This hypothesis is reinforced by experiments in which Pep19-2.5 was added after the virus had already bound to the cells, resulting in an 80% reduction in infection at a concentration of 1 µg/mL. Interestingly, while Pep19-2.5 did not disrupt CD151 accumulations, further imaging revealed large viral aggregates situated away from the plasma membrane in Pep19-2.5-treated cells. This is the first direct visualization of viral release from the cell surface following attachment, suggesting a blockade of the transition from primary HSPG attachment to interaction with the secondary receptor. Previous studies have proposed two non-mutually exclusive models to explain the transition from HSPG attachment to secondary receptor engagement. One model suggests that HSPG binding induces conformational changes in capsid proteins, reducing their affinity for HSPGs and allowing transfer to the secondary receptor (25, 26). The second model suggests that cleavage of HSPGs by matrix metalloproteinases liberates soluble viral complexes, which can then engage the secondary receptor (16). Our findings support a mixed model in which the virus is released by HSPG cleavage, and Pep19-2.5 likely interferes with HPV infection by preventing further interactions with HSPGs, thereby inhibiting the structural activation required for effective binding to the second receptor.

Another study demonstrated that dispirotripiperazine (DSTP) derivative, an HSPG-binding drug, induced the formation of large virus aggregates and non-infectious internalization, further supporting the idea that post-binding interactions to additional HSPG moieties on the cell surface are required for the viral transfer to entry receptor molecules (24). This is in line with our post-binding imaging experiments visualizing again the formation of large virus aggregates that are released from the cell surface. This also supports the biochemical finding in which complexes of capsid proteins and cleaved HSPGs are isolated from cell culture supernatants (16). Although DSTP operates at the same stage as Pep19-2.5, it has a significantly higher IC_50_ in preventing HPV16 infection (24). Similarly, the HSPG-binding peptide SB-105-A10 also exhibits a much higher IC_50_ (38), highlighting Pep19-2.5’s superior effectiveness in preventing HPV infection. In addition, γ-secretase inhibitors demonstrate strong potency with IC_50_ values in the picomolar to nanomolar range in HPV16 infection inhibition (42, 43). Combining Pep19-2.5 with other inhibitors targeting different stages of HPV infection could result in even more effective prevention of infection (44).

The role of Pep19-2.5 in inhibiting virus binding to a non-HSPG molecule is further supported by experiments using CHO cells, where GAG-deficient CHO cells (CHO-ΔGAG) displayed a substantial reduction in HPV infection compared to wild-type cells. The fact that Pep19-2.5 still reduced infection rates in these GAG-deficient cells suggests that its inhibitory effects extend beyond HSPG interactions. This is in line with previous studies in CHO-ΔGAG cells (38) suggesting that positively charged peptides not only block HSPG interactions but also prevent virus binding to alternative, still uncharacterized, receptors. While the second receptor for HPV entry remains unknown, Pep19-2.5 could prove a valuable tool in future studies aimed at identifying this elusive entry receptor.

Furthermore, no additional dose-dependent reduction in HPV16 infection was observed in CHO-ΔGAG cells beyond 1 µg/mL of Pep19-2.5, suggesting that the secondary receptor may saturate quickly, as suggested earlier (23). It remains to be explored whether this secondary molecule influences virus trafficking, modulates signaling pathways necessary for infection, or impacts another aspect of the viral life cycle.

In the context of current HPV prevention strategies, Pep19-2.5 offers a promising alternative or complement to existing measures. While vaccines against HPV have been effective, they are costly and vaccination rates are still low (45, 46). Inexpensive, accessible alternatives like lubricated condoms or topical gels could provide additional preventive strategies. A clinical trial with a carrageenan-based gel, which blocks HPV binding to HSPGs, demonstrated efficacy in reducing genital HPV infections (47). Again, combining Pep19-2.5 with other effective HPV entry inhibitors, such as carrageenan or HPV blocking peptides (48–52), could enhance the effectiveness of topical prevention strategies, especially for individuals without access to vaccines. Importantly, the potency of Pep19-2.5 in post-binding HPV inactivation suggests its potential as a topical microbicide capable of preventing HPV infection during or directly after sexual intercourse.

In conclusion, this study identifies Pep19-2.5 as a promising inhibitor of HPV16 infection, primarily through its interference with virus binding to HSPGs. Additionally, the peptide induces the release of cell-surface bound virus particles, most likely by disrupting viral transfer to secondary receptors. These findings underscore the potential of Pep19-2.5 as a preventative agent against HPV infection and provide insights into the early stages of the unconventional HPV16 entry pathway. Further research will be needed to fully elucidate its exact mechanism of action and optimize its use in clinical settings.

## MATERIALS AND METHODS

### Antibodies, cells, peptides, and pseudoviruses

#### Antibodies

HPV16 L1 mouse monoclonal antibody (mAb) 16L1-312F used in Western blot and rabbit polyclonal antibody (pAb) K75 for imaging experiments have been previously described (17, 53, 54). CD151-specific mouse mAb (11G5a) was obtained from Bio-Rad (Munich, Germany). β-Actin-specific mouse mAb (A5441) was obtained from Sigma-Aldrich (St. Louis, MO, USA). Horseradish peroxidase (HRP)-coupled secondary antibodies for immunoblot were purchased from Jackson ImmunoResearch Europe Ltd. (Cambridgeshire, UK). Secondary antibodies for imaging experiments were Star Green coupled to goat-anti-rabbit (STGREEN-1002-500UG) from Abberior (Goettingen, Germany) and AlexaFluor 594 coupled to donkey-anti-mouse (A21203) from Invitrogen—Thermo Fisher Scientific (Waltham, MA, USA).

#### Cells

The HeLa cell line, derived from human cervical carcinoma, was acquired from the German Resource Center of Biological Material (DSMZ) in Braunschweig, Germany. The HaCaT cell line, consisting of human non-virally immortalized keratinocytes, was obtained from Cell Lines Services in Eppelheim, Germany. The human embryonic kidney cell line (HEK) 293TT used for pseudovirus production was kindly provided by Dr. Christopher B. Buck, Ph.D. (National Cancer Institute, Laboratory of Bethesda, Maryland, USA). In addition, we used the Chinese hamster ovary K1 (CHO-K1, also referred to as CHO-WT) cells and their derivatives pgsA-745 (kindly provided by Martin Sapp, Shreveport, LA, USA) (36). These cells exhibit impaired biosynthesis of heparan and chondroitin sulfates, attributed to a mutation in the xylosyltransferase gene. Cells were grown at 37°C in Dulbecco’s modified Eagle’s medium (DMEM + GlutaMAX) provided by Thermo Fisher Scientific (Waltham, MA, USA), supplemented with 10% fetal calf serum (FCS) from Sigma-Aldrich, 1% minimum essential medium non-essential amino acids (MEM non-essential amino acids) obtained from Thermo Fisher Scientific, and 5 µg/mL ciprofloxacin acquired from Fresenius Kabi (Bad Homburg von der Hoehe, Germany). HeLa cells used for imaging experiments (a kind gift from Waldemar Kolanus, Bonn, Germany) were cultured in high glucose DMEM (Thermo Fisher Scientific) medium supplemented with 10% FCS and 1% penicillin/streptomycin (10,000 U/mL penicillin, 10 mg/mL streptomycin) from PAN Biotech (Aidenbach, Germany). The antibiotic was omitted in the experiments.

#### Peptides

Peptide 19-2.5 (Pep19-2.5; GCKKYRRFRWKFKGKFWFWG) and a control peptide (Do-D; GIGKFSKKGAAARRRKVSLKAL) were synthesized in the Research Center Borstel according to standard procedures (3). Do-D has a similar proportion of hydrophobic, charged, and polar amino acids as the Pep19-2.5.

#### Pseudoviruses

HPV16 PsVs were generated following established protocols (32, 55). HEK293TT cells were seeded into 150 cm^2^ cell culture flasks and transfected with PEI using equal amounts of codon-optimized HPV16 L1/L2 (pShell 16L1/L2wt) and reporter plasmid (pGL4.20 puro HPV16 LCR) for 48 hours. The cells were then detached and centrifuged to pellet the cells. The resulting pellet was resuspended in phosphate-buffered saline (PBS) supplemented with 9.5 mM MgCl_2_ (1× PBS/9.5 mM MgCl_2_), centrifuged again, and resuspended in 1× PBS/9.5 mM MgCl_2_ with 0.5% Brij58 (Sigma-Aldrich) and 0.2% Benzonase (Merck Millipore, Burlington, MA, USA). The mixture was incubated at 37°C on a rotator for 24 hours. Next, the cell lysates were then placed on ice and adjusted to a final NaCl concentration of 0.85 M. After centrifugation, the supernatants were loaded onto iodixanol gradients for PsV purification (Optiprep). The iodixanol gradient consisted of three layers: 39% iodixanol at the bottom, 33% in the middle, and 27% at the top. The gradients were incubated for 90 minutes at room temperature, followed by ultracentrifugation for 3.5 hours at 55,000 rpm and 16°C. Approximately 15 fractions of 300 µL each were collected and analyzed using a luciferase reporter assay to identify peak fractions. PsV quantification was conducted by quantifying the packaged genomes (viral genome equivalents, vge) as previously described (32).

### Infection and LDH assays

For infection assays using cell incubation with peptides prior to PsV addition, cells were seeded in a 24-well plate and allowed to incubate for 24 hours. Following this, the cells were pre-incubated with either the control or Pep19-2.5 for 1 hour at 37°C. Subsequently, the cells were infected with PsVs of approximately 100 vge per cell for another 24 hours. This setting was used, e.g., to determine the concentration required to achieve the half-maximal inhibition (IC_50_).

For infection assays using cell-incubation with peptides post-PsV addition (post-PsV binding), HeLa cells were seeded in a 24-well plate and incubated the following day with PsVs of approximately 100 vge per cell for 1 hour at 37°C. Following PsV incubation, the cells were treated with either the control or Pep19-2.5 at different time points after PsV addition.

Following the incubation period in both settings, the cells were washed once with 1× PBS and then lysed using 1× Cell Culture Lysis Reagent from Promega (Fitchburg, WI, USA). After a brief high-speed centrifugation, luciferase activity in the supernatant was quantified using the LB 942 Tristar 3 luminometer from Berthold Technologies (Bad Wildbad, Germany).

#### LDH assay

Cytotoxic effects are accompanied by a loss of membrane integrity, which can be determined by a loss of LDH in the cell lysate. LDH activity was determined using the CytoTox-ONE Homogeneous Membrane Integrity Assay from Promega. LDH counts in the lysates were assessed according to the manufacturer’s instructions using the LB 942 Tristar 3 luminometer.

### GraphPad analyses

GraphPad Prism 9 for Windows (version 9.4.1., GraphPad Software, San Diego, CA, USA) was utilized for data analysis.

#### Half-maximal inhibitory concentration (IC_50_)

For IC_50_ determination, luciferase counts normalized as percentages were logarithmically transformed with the base of the logarithm set to 10. The transformed data underwent analysis using nonlinear regression (curve fit): dose-response − inhibition; log(inhibitor) vs normalized response − variable slope.

#### Statistics

Statistical differences between the groups were assessed using Welch’s *t* test. Graphs represent at least three biological replicates, with two technical replicates from each biological replicate. The graphs display a mean ± standard error of the mean (mean ± SEM) for all data points (technical replicates). Exact *P* values are given and stated in the figure legend for each statistical test. The “*n*” (specified in the figure legend) denotes the number of data points (technical replicates) per group collected from independent biological replicates. Differences between the groups were considered statistically significant when *P* ≤ 0.05 with the levels of statistical significance marked in the graph (*P ≤ 0.05, **P ≤ 0.01, ***P ≤ 0.001, *P* > 0.05, ns = not significant).

### Binding assay

HeLa cells were grown in 24-well plates and subjected to treatment with either the control or Pep19-2.5 for 1 hour at 37°C. Subsequently, the cells were exposed to PsVs of approximately 100 viral genome equivalents (vge) per cell for 2 hours at 4°C. Following this, the cells underwent washing with 1× PBS to eliminate any unbound PsVs. Finally, the cells were harvested in sodium dodecyl sulfate (SDS) sample buffer for Western blot analysis.

### Western blot analysis

Cells were rinsed with PBS, then lysed in SDS sample buffer containing 10% 2-mercaptoethanol and denatured at 95°C for 5 minutes. Equal amounts of protein were loaded onto an SDS-polyacrylamide gel (SDS-PAGE). Subsequently, the proteins were electro-transferred onto Amersham Protran 0.45 µm nitrocellulose membrane from Cytiva (Marlborough, MA, USA) and blocked with 5% milk powder in PBS containing 0.1% Tween-20 (PBS-T). Following this, the membrane was incubated with primary antibody at 4°C overnight. The next day, the membrane was washed in 1× PBS supplemented with 0,1% Tween (PBS-T) and incubated with HRP-conjugated secondary antibodies for 1 hour at room temperature. Signal detection was carried out using the Western Lightning Plus-ECL detection reagent from PerkinElmer (Waltham, MA, USA). Chemiluminescent signals were recorded with iBright1500 documentation system from Thermo Fisher Scientific. Densitometric analysis was performed using ImageJ 1.54f software (Wayne Rasband and contributors, National Institutes of Health, USA, http://imagej.org).

### Sample preparation for imaging of post-PsV binding experiments

HeLa cells were plated onto PLL (poly-L-lysine)-coated glass-coverslips in six-well plates and incubated for 24 hours. The next day, cells were incubated with PsVs of approximately 130 vge per cell for 1 hour at 37°C. An hour after PsVs addition, 1 µg/mL control peptide (ctrl.), Pep19-2.5 (pep.), or no peptide was added (−pep.). After an additional 3 hours, cells were washed in PBS and fixed with 4% paraformaldehyde (PFA) in PBS for 30 minutes. PFA was removed, and residual PFA was quenched by 50 mM NH_4_Cl in PBS for 30 minutes, gently shaking the plates. Afterward, the samples were blocked with 3% bovine serum albumin (BSA) in PBS for 30 minutes. Omitting any detergents, surface L1 and CD151 were stained using the pAb K75 against L1 (diluted 1:1,000) and mAb against CD151 (diluted 1:200) in 3% BSA-PBS for 2 hours. Cells were washed three times with PBS, and secondary antibody staining was performed using Star Green goat-anti-rabbit (diluted 1:200) and AlexaFluor 594 donkey-anti-mouse (diluted 1:200) in 3% BSA-PBS for 1 hour. Finally, samples were washed three times with PBS and mounted on microscopy slides using ProLong Gold antifade mounting medium (Invitrogen).

Confocal and STED micrographs were recorded employing a 4-channel STED microscope from Abberior Instruments (available at the LIMES Institute imaging facility, Bonn, Germany). The microscope is based on an Olympus IX83 confocal microscope equipped with a UPlanSApo 100× (1.4 NA) objective (Olympus, Tokyo, Japan). For *x*,*y*-scans at a pixel size of 25 nm, we combined confocal imaging of PsVs (the PsVs density does not necessarily require STED resolution; we used a 485 nm laser for Star Green excitation, and emission was detected at 500–550 nm) and STED imaging of CD151 (using a 561 nm laser for excitation of AlexaFluor 594 in combination with a 775 nm laser for depletion and emission was detected at 580–630 nm). The focal plane was adjusted to the cell base. Per condition and biological replicate, 15 images were recorded.

For *x*,*z*-scans and *y*,*z*-scans, we employed confocal imaging using a 485 nm laser for Star Green excitation detecting emission at 500–550 nm and a 561 nm laser for AlexaFluor 594 excitation detecting emission at 580–630 nm. Pixel size was set to 25 nm for the *x* and *y* dimensions, with a variable pixel size of the *z* dimension, ranging from 134 to 231 nm, depending on the size of the recorded image. In this experiment, we searched specifically for cells exhibiting large accumulations of PsVs. With a single exception shown in Fig. S1 (control peptide condition, ctrl.), such examples could be only found in the condition with Pep19-2.5, in a minority of the cells. Per condition and biological replicate, 20 images were recorded.

Images were analyzed using Fiji ImageJ in combination with a custom-written macro, essentially as described previously (56). Prior to analysis, images (*x*,*y*-scans) were smoothed with a Gaussian blur (*σ*  =  0.5) to reduce pixel noise and improve maxima detection. Then, aiming for sampling all aspects of the largely variable local L1 (PsVs) and CD151 signal density, we defined rectangular ROIs such that ROIs covered roughly half of the cell base, including areas of high and low CD151 density.

L1 and CD151 maxima were detected using the “Find Maxima” function (noise tolerance 4 for CD151 and 150 for L1), yielding L1 and CD151 maxima positions in pixel positions. For analyzing whether the peptide reduces the number of CD151 maxima at PsV positions (detected as PsV maxima), CD151 maxima close to L1 maxima were counted. To this end, at every L1 maxima position, a 925 nm circular ROI was placed, and the number of CD151 maxima in this ROI was counted employing the same noise tolerance as above. For each cell, the counted CD151 maxima at every L1 position were normalized to the average CD151 maxima density of the large rectangular ROI, yielding the normalized L1 neighbored CD151 maxima.

To obtain a control for the randomized association between L1 and CD151, using the same L1 maxima positions, the same procedure was employed on horizontally and vertically flipped ROI areas of the CD151 channel. In flipped (randomized) data, plotting frequency against the number of normalized L1 neighbored CD151 maxima, one obtains a Gaussian-shaped distribution peaking at a value of 1 (see also Fig. 4B, lower panels). The non-randomized distribution is more right shifted (see also Fig. 4B, top panels), as it contains next to the L1 maxima with random neighbored CD151 maxima as well L1 maxima with an elevated number of neighbored CD151 maxima. The two fractions are obtained by decomposition of the distribution (continuous lines in Fig. 4B) into two Gaussian distributions (Fig. 4B, dotted and dashed lines, the gray dotted-dashed lines are the sum of both).

In the same large rectangular ROIs, the PCC between L1 and CD151 was calculated with a custom-written ImageJ macro.

In *x*,*z*-scans and *y*,*z*-scans, to analyze the fraction of distal PsVs, the images of both channels were interpolated in the *z* dimension by a factor of 8. Then, the cell was outlined with reference to the CD151 signal (for example, ROIs, see Fig. S2A). Using these ROIs, we measured the integrated density of L1 in the cell outline. In addition, the integrated density of the entire image was measured. For background correction, we placed a small ROI to a background region, measured the integrated density, and calculated with reference to the area of the cell outline and the entire image, the included background signal that was subtracted. From the background corrected values, we calculated the percentage of distal PsVs as 100% (entire image) − percentage in the “cell outline.”

## SUPPLEMENTAL MATERIAL

Supplemental material aac.01575-24-s0001.docx Figures S1 and S2.

ASM does not own the copyrights to Supplemental Material that may be linked to, or accessed through, an article. The authors have granted ASM a non-exclusive, world-wide license to publish the Supplemental Material files. Please contact the corresponding author directly for reuse.
