# Supplementary material for: HSPG-binding peptide Pep19-2.5 is a potent inhibitor of HPV16 infection
Source: Antimicrob Agents Chemother. 2025 Jan 14;69(2):e01575-24. doi: 10.1128/aac.01575-24 (PMC11823620; doi:10.1128/aac.01575-24)
Supplement: Supplemental material — Figures S1 and S2. [file aac.01575-24-s0001.docx]

**
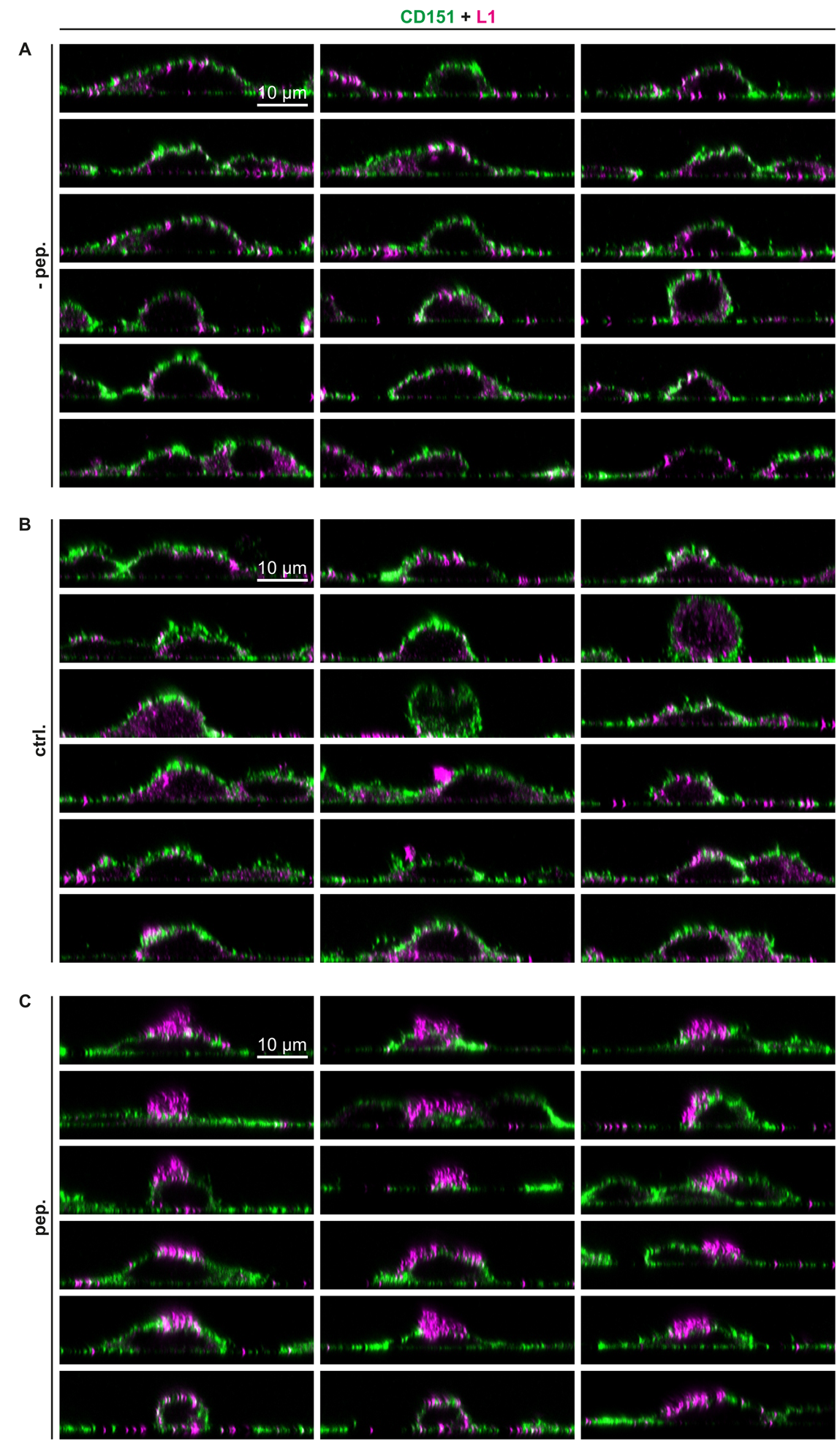
**

**Fig. S1. Variation in the sample of the experiment shown in Figure 5.** For one replicate analyzed in Figure 5, we show the first 18 of the 20 recorded images for each condition as overlays, employing auto-scaling of the L1 (magenta) and CD151 (green) channels. (A) Without any peptide (-pep.), (B) with control peptide, and (C) with Pep19-2.5.

**
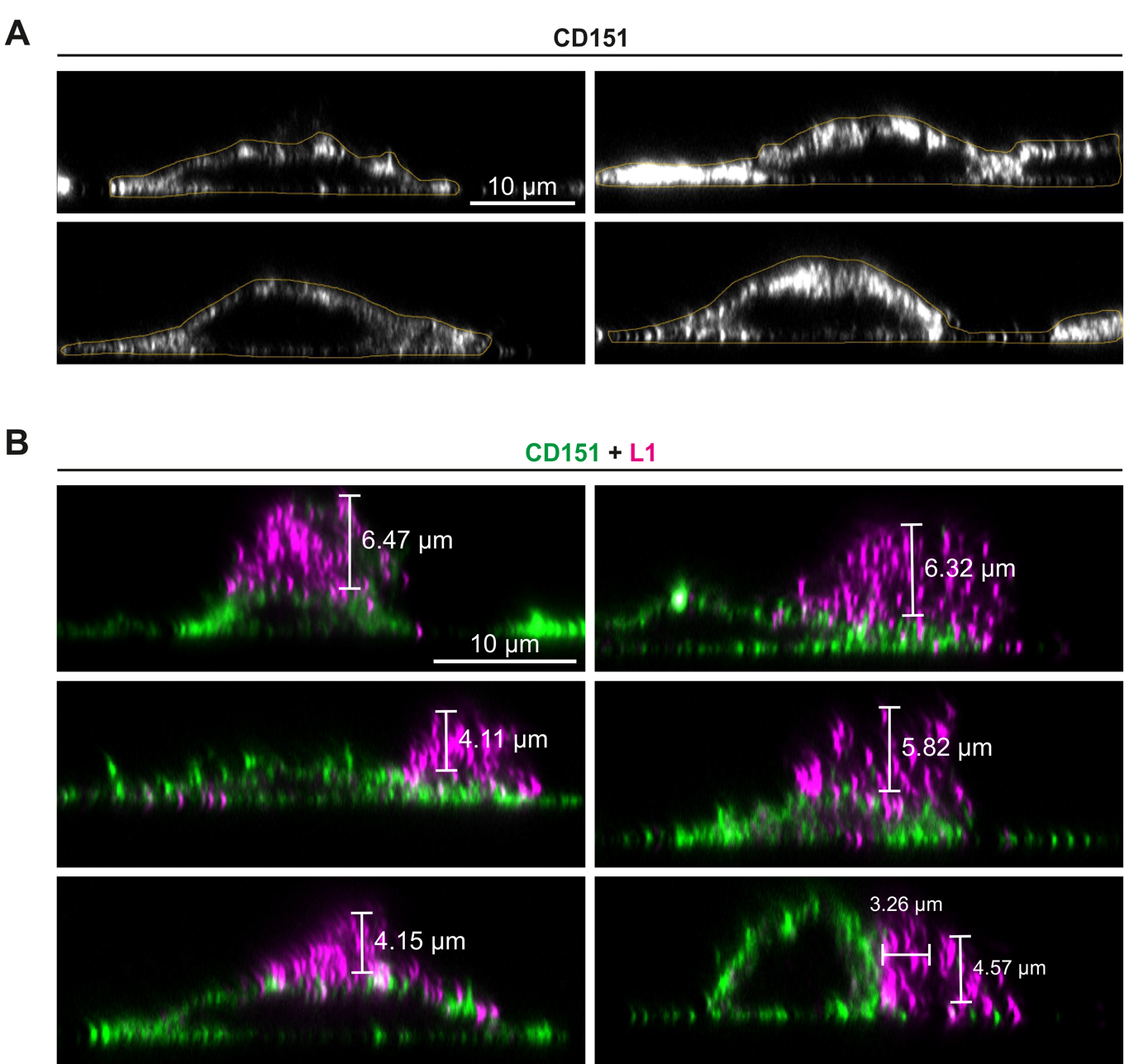
**

**Fig. S2. Example outline used for quantification of distal PsVs and largest distances between PsVs and the cell surface.** (A) Illustration of outlines used for quantification of the distal PsV fraction. Shown are auto-scaled images. (B) Representative images collected from all three replicates of the Pep19-2.5 condition. Overlays of auto-scaled images are shown. Bars illustrate the distance between the most distal PsVs and the cell surface, as indicated by the CD151 cell-surface staining.
